# Comparison of the push-out bond strength of AH Plus sealer to dentin after using different herbal irrigation solutions as the final rinse

**DOI:** 10.1371/journal.pone.0276666

**Published:** 2022-11-02

**Authors:** Mohammadreza Nabavizadeh, Fereshte Sobhnamayan, Mahdi Sedigh-Shams, Sepideh Liaghat

**Affiliations:** 1 Department of Endodontics, Oral and Dental Disease Research Center, School of Dentistry, Shiraz University of Medical Sciences, Shiraz, Fars, Iran; 2 Department of Endodontics, School of Dentistry, Shiraz University of Medical Sciences, Shiraz, Fars, Iran; Nair Hospital Dental College, INDIA

## Abstract

The aim of the present study was to evaluate the push-out bond strength of AH Plus sealer to dentin treated with the essential oils of Cumimum cyminum and Cinnamomum zeylanicum as herbal final irrigants. Forty human mandibular first premolars were selected. After smear layer removal, the samples were divided into 4 groups and irrigated with experimental solutions for 1 min and later with distilled water. G1: Cinnamomum zeylanicum (CZ) in minimum inhibitory concentration (MIC); G2: Sodium hypochlorite 2.5%; G3: Sodium hypochlorite in MIC; G4: Cuminum cyminum (CC) in MIC. After obturation, the roots were sectioned in order to obtain 1-mm discs for push-out assessment. The push-out test was performed using a universal testing machine. The slices were examined using a stereomicroscope at 30× to determine the mode of failure. The data were analyzed using one-way analysis of variance and Tukey’s post-hoc test. The teeth irrigated with CZ showed significantly lower push-out resistance than those irrigated with NaOCl 2.5% and NaOCl at MIC. The other groups had no significant difference. The modes of failure were predominantly mixed. Under the limitations of the present study, CC does not have adverse effects on the bond strength of AH Plus and can be used as a good alternative for currently used final irrigants.

## Introduction

A successful endodontic treatment depends on a thorough chemomechanical preparation of the root canal system as well as a three dimensional filling with an impervious, biocompatible, and dimensionally stable filling material [1].

Gutta-percha alone cannot adhere to the root canal dentin. Thus, sealers are used to bond it to the root canal dentin [2]. Several kinds of sealers are available for endodontic use from early introduced ZOE-based sealers to epoxy resin-based sealers. Epoxy resin-based sealers such as AH Plus are slowly replacing other contemporary sealers due to their desirable physical properties, reduced solubility, adequate biological performance, better sealing ability, and improved micro-retention to root dentin [3].

Furthermore, chemical irrigants are necessary for a thorough root canal disinfection, lubrication of the dentinal wall, flushing out of debris [4], dissolving organic and inorganic debris [4], and improving the bonding ability of resin-based sealers [5]. Sodium hypochlorite is the most widely used irrigant due to its strong antibacterial activity as well as its ability to dissolve organic materials and remove necrotic tissues [6].

The adverse effects of NaOCl have been reported as unpleasant odor and taste, toxicity, possible paresthesia of the mandibular nerve, allergy, degradation of dentin by the dissolution of collagen, and an increase in coronal microleakage of adhesive restorations [7–9]. It has been proved that NaOCl inhibits the polymerization of AH Plus sealers when used as a final irrigant [10].

The side effects of synthetic drugs have prompted researchers to look for herbal alternatives. Herbal products used in endodontics have several advantages such as low cost, ease of use, and increased storability [11]. Medicinal herbs are supposed to be potential sources of bioactive compounds [12]. Some natural plant extracts are effective in the treatment of infectious diseases. They are biocompatible and mitigate the side effects of synthetic antimicrobials, suggesting that they can be potentially used as endodontic irrigants and intracanal medications [13]. Furthermore, most herbal irrigants are safe and nontoxic to host tissues [14].

Recent researches have focused on herbal alternatives to defeat resistant microorganisms harboring in the root canal system. Ramazan et al. showed that the biosynthesized AgNPs extracted from wild ginger exhibited complete antibacterial activity against multidrug resistant bacteria [15]. A recent study showed the anti-inflammatory characteristic of traditional medicinal herbs in preventing and controlling oral disease conditions such as gingivitis and periodontitis through protease inhibition activity [16]. The antimicrobial activity of some herbs such as Cinnamomum zeylanicum [17] and Cuminum cyminum has been proved in recent studies [18]. Zingiber officinale showed an effective antibacterial properties against gram positive bacteria. Furthermore it has been considered as a potential natural source of antioxidants [19]. Cuminum cyminum also has excellent antifungal [20–22] and analgesic properties [23]. Two recent studies showed that cumin essential oil was a more potent antimicrobial agent compared to CHX against aerobic bacterial mixture, anaerobic bacterial mixture and E. faecalis and related this antimicrobial activity to the presence of cumin aldehyde and other major components in the composition of this essential oil [24, 25]. Kangabam et al. showed that, methanol, ethanol, isopropyl alcohol, acetone, and chloroform extracts of Cinnamomum zeylanicum were found to be effective antibacterial agents against E. faecalis—both planktonic cells and 6 weeks biofilm formed on dentin substrate [26]. Thus, the use of these essential oils could be considered as an alternative agent for antimicrobial therapy. Nabavizadeh et al. also showed that the surface tension of these plants was optimal and better than that of NaOCl and these essential oil were able to decrease the contact angle between AH 26 sealer and dentin surface [27]. The bond strength of root end filling material is dependent upon both material properties and the surface of root end preparation [28]. The physicochemical reaction between root end filling materials and dentin results in an adhesion reaction between them [29, 30]. The bond strength of a material with dentin is a significant factor for the success of the various endodontic procedures, therefore, push-out test methods have been developed to assess this property of restorative materials [29, 30]. This essential oil as an irrigant might result in better binding of sealers into dentinal walls [27]. Considering the superior effect of these essential oils in antimicrobial activity and wettability compared to NaOCl and CHX, and Nabavizade et al. also showed that the surface tension of these plants was optimal and better than that of NaOCl [27].

Since irrigants can affect the bond strength of resin sealers [31–33], the current study was conducted to evaluate the effect of using herbal extracts (Cinnamomum zeylanicum [CZ] and Cuminum cyminum) as irrigation solutions on the push-out bond strength of AH Plus sealer.

## Materials and methods

This in-vitro study was approved by the Ethics Committee of Shiraz University of Medical Sciences (IR.SUMS.REC.1396.S172) and since the samples were the teeth extracted for other reasons, the ethics committee waived the need for consent.

### Irrigant preparation

**Fig 1:**
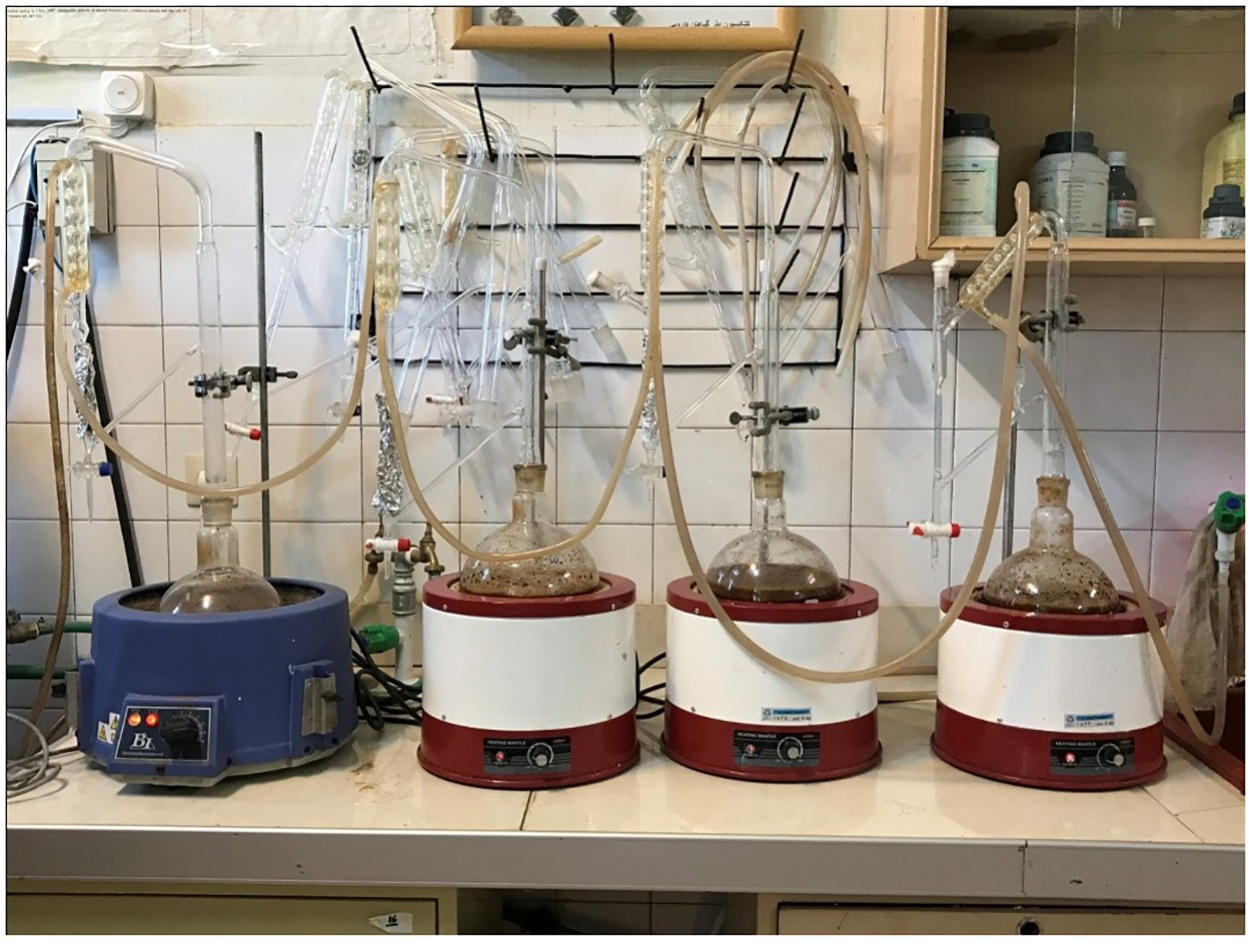
Clevenger type apparatus.

800 gram seeds of cumin from Joupar region in Kerman province, Iran and 800 gram Cinnamomum zeylanicum barks were purchased from a local medicinal plant store in Shiraz and then identified and authenticated an expert plant taxonomist, based on morphological depiction and regarding the known samples that have been previously collected. For CZ a voucher specimen (Number 666- Cinnamomum zeylanicum) and for CC a voucher specimen (Number 1407- Bunium persicum (Boiss.) B.Fedtsch.) has been deposited at Herbarium of the Department of Pharmacognosy, Shiraz School of Pharmacy. Plants were washed and stored in a sheltered place for 20 days at room temperature and air-dried. To provide the pertinent form of the CC and C.Z, a blender ground the plants to produce a fine powder. To provide the essential oil, 300 gm of these powders were steam distillated by using a Clevenger-type apparatus (Dorsa, Iran) (yield: 0.93%±0.23) (Fig 1). The organic layer was parted, then concentrated under pressure, dried over anhydrous sodium sulfate (2.5 mg/ ml concentration), and finally stored in sealed vials at low temperature (4°C). The essential oils of the plants were obtained by steam distillation using a Clevenger-type apparatus (Fig 1). Primary concentration of CC was 289550 μg/ml and for CZ was447500 μg/ml. Then, they were diluted by dimethyl sulfoxide to obtain their MICs (minimum inhibitory concentrations) (CC:103 ×36μg/ml and CZ: 103 ×14μg/ml).

### Specimen preparation

Mandibular premolars were selected from a collection of teeth that had been extracted for reasons unrelated to this study. The specimens were immersed in 0.5% chloramine T solution (Merck, Darmstadt, Germany) for 48 hours in order to disinfect the surface of the teeth. They were then stored in distilled water until use. The soft tissue and calculus were removed mechanically from the root surfaces with a periodontal scaler. The teeth were radiographically imaged to verify that they had a single root canal without calcification. The exclusion criteria consisted of teeth with more than a single root canal and apical foramen, previous root canal treatment, internal/external resorption, immature root apices, caries/cracks/fractures on the root surface, and/or root canal curvature of more than 10 degrees.

According to the aforementioned criteria, 40 mandibular premolar teeth with similar root lengths from the cementoenamel junction to the root apex were selected. The specimens were decoronated using a diamond disk to acquire a standardized root length of 15 mm. A size 10 K-file (Dentsply Maillefer, Ballaigues, Switzerland) was placed in the canal until it was visible at the major apical foramen. The working length was determined by subtracting 1 mm from this measurement.

The root canals were prepared using ProTaper rotary instruments (Dentsply Maillefer) up to F4 (size 40, 0.06 taper). The root canals were irrigated with 2 mL of 2.5% sodium hypochlorite (NaOCl) (Cerkamed CHLORAXID, Poland) between the instrument changes. Then, each sample was treated with 3 mL of 17% EDTA (Ethylene diamine tetraacetic acid) for 5 min followed by 5 mL of NaOCl 5.25% for 5 min to remove the smear layer. All specimens were rinsed with 2 ml of distilled water according to Table 1.

**Table 1:** Experimental groups and protocol of irrigation.

Groups	Smear layer removal	Intermediate rinse	Final rinse treatment	
1	5 ml NaOCl (5.25%)+ 3 ml 17%EDTA 5 min	2 ml Distilled water (1 min)	10 ml Cinnamomum zeylanicum (1 min)	2 ml Distilled water (1 min)
2	5 ml NaOCl(5.25%)+3 ml 17%EDTA 5 min	2 ml Distilled water (1 min)	10 ml hypochlorite 2.5% (1 min)	2 ml Distilled water (1 min)
3	5 ml NaOCl(5.25%)+3 ml 17%EDTA 5 min	2 ml Distilled water (1 min)	10 ml hpocholorite (MIC) (1 min)	2 ml Distilled water (1 min)
4	5 ml NaOCl (5.25%) + 3 ml 17% EDTA 5 min	2 ml Distilled water (1 min)	10 ml Cuminum cyminum (MIC) (1 min)	2 ml Distilled water (1 min)

Finally, they were irrigated with 2 mL of distilled water [32] for 1 min.

The specimens were dried using paper points (Puma Dent, China). A single gutta-percha cone (F4, Dentsply Maillefer) slightly coated with AH Plus sealer (Dentsply DeTrey,Konstanz,Germany) was placed in the root canal to the working length. Because the root canals were prepared using rotary instruments up to F4 files, all specimens were obturated using the single technique to obtain standard specimens for the push-out test [34].

**Fig 2:**
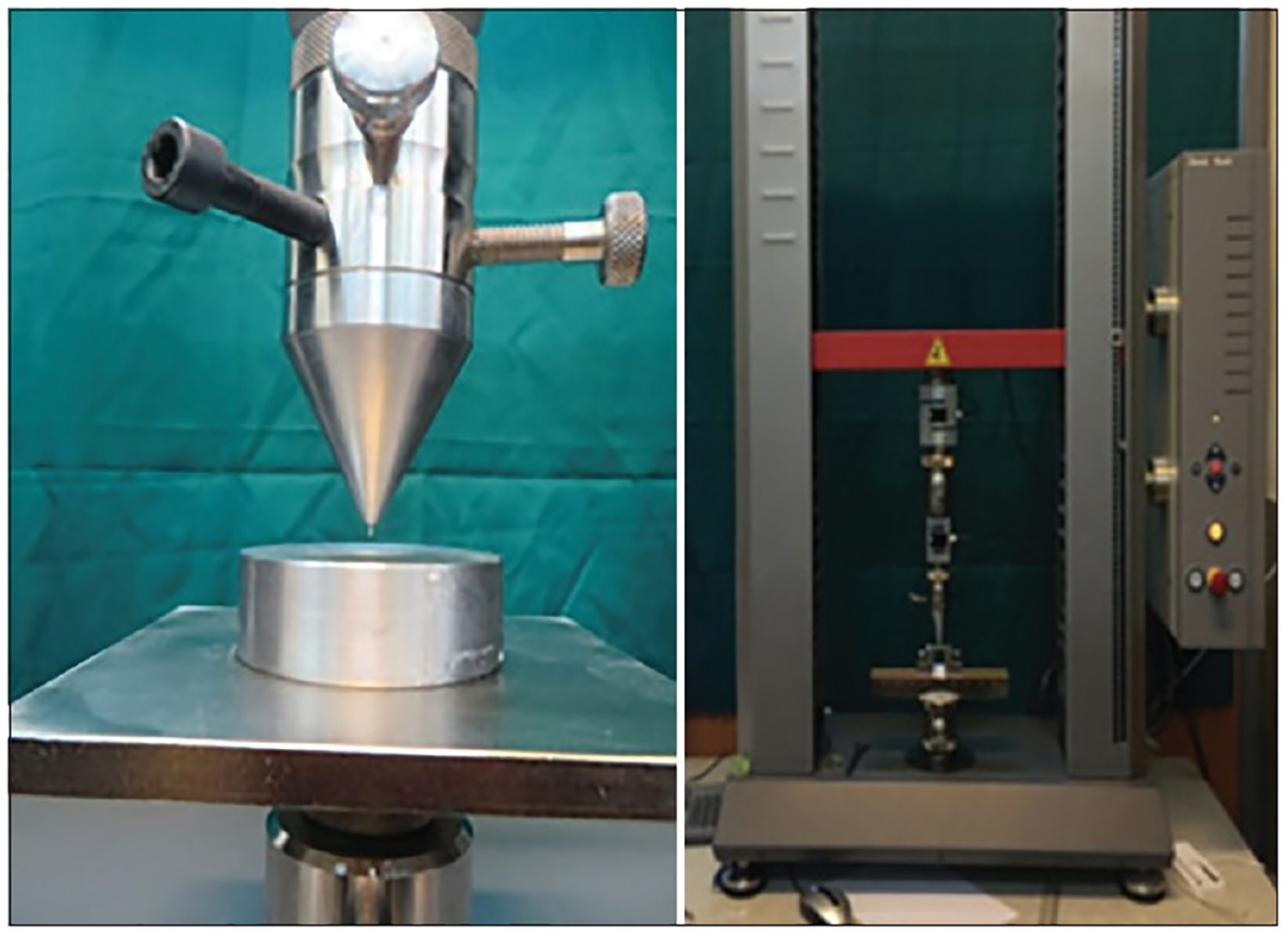
Universal testing machine.

Afterward, the coronal opening was filled with a temporary filling material (EX Temp,paria,Iran) and the specimens were stored in 100% humidity at 37°C for 1 week in order to set completely [35]. All procedures have been performed with a single operator. All specimens were sectioned perpendicular to their long axis using a precision saw (Mecatome T180, Presi, France) at a low speed under water cooling. Two slices were obtained from the midroots of each tooth (n = 20) with a thickness of approximately 1.5±0.1 mm. The thickness of each slice was measured using a digital calliper (Teknikel, Istanbul, Turkey) to an accuracy of 0.001 mm. Sixteen specimens with round canals were chosen from these disks. The diameter of each hole from the apical and coronal aspects was measured under a stereomicroscope (Microscope, Best Scope, China) at 32× magnification. The push-out test was performed on each specimen with a universal testing machine (ZOZO, Zwick/Roell, Germany) at a crosshead speed of 1 mm/min using 0.7-mm diameter cylindrical plungers based on the diameter of the canal (Fig 2). The diameter of the plungers was approximately 80% of the diameter of the canal. The maximum load applied to the filling material before failure was recorded in Newtons and converted to Megapascals [36] according to the following formula:

$$\text{Push-out}\;\text{bond}\;\text{strength}=\frac{N}{\pi \left(\;r1+r2\right)(\sqrt{{\;(r1-r2)}^{2}+h^{2})}}$$

**Fig 3:**
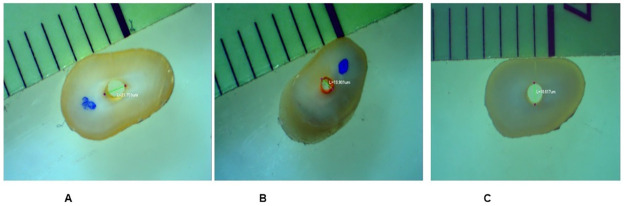
Failure type A) mixed, B) cohesive, C) adhesive.

r1 and r2 are respectively the smaller and larger radii of the canal diameter (mm), h represents the thickness of the root section (mm), and π is the constant 3.14 [37]. After the test procedure, each specimen was visually examined under a stereomicroscope at 32× magnification to evaluate the failure type. Three types of failure were categorized: adhesive failure (between the sealer and root dentin), cohesive fracture (within the sealer or root dentin), and mixed (a combination of cohesive and adhesive) (Fig 3) [38].

The data were statistically analyzed using one-way analysis of variance (ANOVA) followed by Tukey’s post-hoc test with SPSS 11.0 software. The significance level was set at 0.05.

## Results

**Fig 4:**
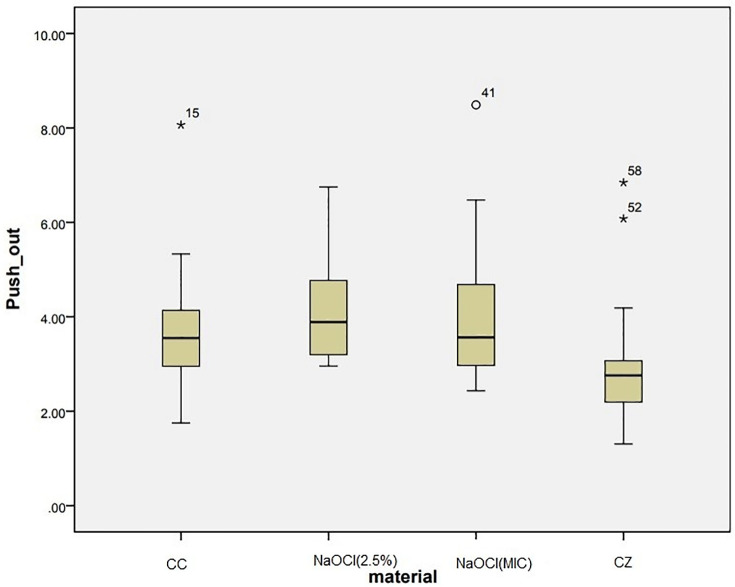
Push out bond strength of experimental groups.

As displayed in Table 2, one-way ANOVA test showed a significant difference between the groups (P = 0.001). Tukey’s post-hoc test showed that CZ showed significantly lower push out bond strength than other groups (P = 0.01). There was no significant difference in push out bond strength of CC and NaOCl groups {2.5% NaOCl (P = 0.665) and NaOCl at MIC (P = 0.116)} (Fig 4). In addition, there was no significant difference in push out bond strength of NaOCl at MIC and 2.5% NaOCl (P = 0.903) (Table 3). The analysis of the failure modes showed the predominance of mixed failures in all groups (Table 2). The teeth irrigated with CZ had significantly lower push-out resistance than those irrigated with NaOCl 2.5% and NaOCl at MIC. The other two groups showed no significant difference.

**Table 2:** The mean ± standard deviation of the push-out bond strength of each group.

Groups	Cuminum cyminum	NaOCl 2.5%	NaOCl (MIC)	Cinnamomum zeylanicum
Mean±SD	3.43 ±1.01^a^	4.1±1.04^a^	3.85±1.25^a^	2.57±0.72^b^

*Different superscript lowercase values (a, b) indicate statistically significant differences between the groups (*P* ≤ 0.05).

**Table 3:** Frequency of different types of failure modes in the groups (%).

Fracture mode	Cohesive	Adhesive	Mixed
Group			
Cuminum cyminum	6.8	26.6	66.6
Cinnamomum zeylanicum	0	20	80
NaOCl 2.5%	6.7	20.1	73.2
NaOCl	6.7	13.3	80

## Discussion

In recent years, there has been growing interest in the use of herbal irrigation solutions with pharmaceutical properties. Because of the cytotoxicity of most irrigation solutions and their direct contact with tissues, there is a great tendency to use biologic medication extracted from natural plants in endodontic treatment [39]. Previous studies which compared the antimicrobial activities of different irrigation solutions used different concentrations of NaOCl from 0.5 to 5.25 [40, 41]. Controversial data has been found in different studies about the antimicrobial effect of herbal irrigants. Two recent studies showed that cumin essential oil was a more potent antimicrobial agent compared to CHX against all groups of microorganisms [24, 25].

Kangabam et al. showed that cinnamon extracts can be an effective alternative to NaOCL [26]. Teja et al. in a systematic review revealed that herbal agents showed less efficiency than different concentrations of sodium hypochlorite regarding the antimicrobial property [42]. This controversy in the results of different articles could be attributed to different type of herbal irrigants, and the composition of herbal essential oils which are variable depending on several factors such as geographic region, harvest time, extraction method and the type of culture.

In the present study, NaOCl 2.5% and NaOCl at MIC were used. In clinical studies, the concentration of 2.5% is also acceptable since no significant difference in the antimicrobial activities of different concentrations of NaOCl solution in infected root canals has been shown. On the other hand, in the infected canal, the host defense mechanisms are less active and success is more dependent on the antimicrobial properties of the irrigant. The determination of MIC is important because it shows the extent of antimicrobial activity [12, 43, 44]. Therefore, the MIC of NaOCl was compared with that of herbal extracts [12, 45].

Herbal oils are potential sources of antimicrobial compounds. These essential oils are hydrophobic. Hence, they can degrade the lipids of the bacterial cell wall and mitochondria and thus destroy bacterial structures [46].

The aldehyde and ketone components of essential oils determine the level of their antimicrobial activity [47]. Furthermore, several researchers have shown that terpinen, pinene, and cymen are responsible for the biological effects of essential oils [48].

The reasons to select these plants in the present study were their antimicrobial activity and acceptable wetting ability [12]. Previous studies have shown that CC has promising antibacterial, antifungal, and antioxidant activities [49, 50].

The reasonable antibacterial activity of the essential oil of CC against some Gram-positive and Gram-negative bacteria has been proved [49, 51, 52].

Nabavizadeh et al. [27] showed that CC had a lower contact angle than normal saline and sodium hypochlorite. Furthermore, CZ showed an acceptable wetting ability and contact angle similar to sodium hypochlorite. However, it had a significantly higher contact angle than CC.

The results of the present study showed that CC did not decrease the bond strength of AH Plus sealer and had the same value of adhesion as NaOCl at 2.5% and MIC.

On the other hand, this value was decreased for CZ. Further studies are needed to understand the mechanism by which CZ reduces the push-out bond strength of resin sealers to dentin. The essential oils of both CC and CZ are hydrophobic and could negatively affect the bond strength of resin sealers. However, it seems that the great antioxidant properties of CC make it a better alternative to CZ [53]. The treatment of dentin surfaces with antioxidants enhanced the bond strength of some resin posts [54]. Similarly, some antioxidant agents enhanced the push-out bond strength of AH Plus sealer to root dentin [36, 55]. Using these antioxidant agents as the final irrigation also compensates the negative effect of NaOCl on the bond strength of AH Plus [36]. However, this is not the case with all antioxidant irrigants and sealers [56, 57].

Like NaOCl, CZ has an oxygenated component such as trans-cinnamaldehyde. This may negatively affect the push-out bond strength of resin sealers [58, 59]. The hydrophobicity of CC may reduce the bond strength of the sealer. However, in this study, the bond strength of the AH Plus sealer did not significantly change when CC or NaOCl were used as irrigants. The reason may be related to the antioxidant features of CC that compensate the above-mentioned negative feature. Hydrophobicity and oxidation are the negative features of CZ. This explains the poor results of NaOCl and CC.

The better result of CC compared to CZ in the present study could also be attributed to the carbonyl groups in CC which can provide a great diversity of possible modification for surfaces, increase the wetting ability, and thus increase the interaction between the dentin surface and this irrigant [60]. This causes a better penetration of the sealer into the dentinal tubules and thus increases the push-out bond strength of these samples compared with the CZ group. The lower bond strength in the CZ group could be attributed to its higher contact angle than that of the CC group which could not wet the surface of the dentin [27]. This could result in less contact of the irrigant with the dentin surface and thus less penetration of the sealer into the dentinal tubules.

Regarding the action of sodium hypochlorite in the NaOCl groups, the dentin bond strength after NaOCl treatment has been attributed to its deproteinizing action. NaOCl has the ability to dissolve and remove the exposed dentinal collagen and provide a fresh mineralized dentin surface to which the adhesive resin can be applied. This allows a direct adhesion between the adhesive resin and dentin without the resin-reinforced collagen layer called the ‘hybrid layer’ [61]. The adhesive resin will therefore infiltrate the mineralized matrix filling the submicron porosities. This creates a layer of resin-infiltrated mineralized matrix [61].

There are only few studies that compared the push out bond strength of herbal irrigation with conventional endodontic irrigants. Al Azzawi et al. showed that the bond strength of iRoot SP sealer to dentin samples treated with herbal extracts (green tea and Salvadora persica) in the midroot was significantly greater than waterlase group [62]. On the other hand Choudhury et al in an invitro study showed that Chitosan and Morinda citrifolia juice (MCJ) could increase sealer penetration and prevents the dislocation of obturating materials although EDTA was more efficient in smear layer removal. Results of this study showed that EDTA had the highest pushout bond strength when compared to MCJ and chitosan solution [32]. In another study Shweta compared the push out bond strength of AH plus sealer to dentin when irrigated with Azadirachta indica, Curcuma longa, methyl ethylene diaminotetraacetic acid, and sodium hypochlorite (NaOCl) as irrigating solution. In the midroot part NaOCL samples showed the greatest push out bond strength followed by Azadirachta indica, MTAD and Curcuma longa. But in the cervical part, MTAD showed the greatest bond strength followed by A. indica and C. longa [63].

These controversial results could be attributed to the different methods have been used in these studies, different herbal irrigants used and different synthetic irrigation solutions.

In the present study, the treatment of root canal dentine with essential oils may leave residues on dentine surfaces, reducing the interfibrillar spaces that serve as diffusion channels for infiltration of resin-base sealers in some dentin surfaces resulting in predominance of mixed failure types.

The authors of this in-vitro study put forth the standpoint that these herbal irrigants might affect the microhardness of radicular dentin and the results do not translate the clinical scenario, However, future clinical studies can focus on the use of these herbal agents in attaining optimal disinfection.

## Conclusions

Under the limitations of the present study, CC can be used as a good alternative for currently used final irrigants since it does not have adverse effects on the bond strength of AH Plus.

## Supporting information

S1 Table Raw data of compressive strength of innamomum zeylanicum group. (XLS)

S2 Table Raw data of compressive strength of Cuminum cyminum group. (XLS)

S3 Table Raw data of compressive strength of NaOCl 2.5% group. (XLS)

S4 Table Raw data of compressive strength of NaOCl MIC group. (XLS)
